# Investigating the Influence of Intergroup Contact in Virtual Reality on Empathy: An Exploratory Study Using AltspaceVR

**DOI:** 10.3389/fpsyg.2021.815497

**Published:** 2022-02-02

**Authors:** Matilde Tassinari, Matthias Burkard Aulbach, Inga Jasinskaja-Lahti

**Affiliations:** Faculty of Social Sciences, Department of Social Research, University of Helsinki, Helsinki, Finland

**Keywords:** intergroup contact, virtual reality, empathy, social psychology, intergroup relations

## Abstract

Virtual Reality (VR) has often been referred to as an “empathy machine.” This is mostly because it can induce empathy through embodiment experiences in outgroup membership. However, the potential of intergroup contact with an outgroup avatar in VR to increase empathy is less studied. Even though intergroup contact literature suggests that less threatening and more prosocial emotions are the key to understanding why intergroup contact is a powerful mean to decrease prejudice, few studies have investigated the effect of intergroup contact on empathy in VR. In this study, we developed a between-participants design to investigate how VR can be used to create a positive intergroup contact with a member of a stigmatized outgroup (ethnic minority) and present the results of the effect of intergroup contact in VR on empathy. Sixty four participants experienced either positive contact (i.e., equal intergroup status, collaborative) with a black (experimenter-controlled) avatar (experimental condition) or no intergroup contact (i.e., ingroup contact with a white avatar; control condition), with situational empathy (personal distress and empathic interest) being measured through a self-report questionnaire up to a week before and right after the VR contact experience. The experiment showed that satisfying degrees of body ownership of participants’ own avatar and co-presence with the contacted avatar can be achieved in simple and universally accessible virtual environments such as AltspaceVR. The results indicated that while VR intergroup contact had no significant direct effect on empathy, exploratory analyses indicated that post-intervention empathic interest increased with stronger feelings of co-presence in the intergroup contact condition.

## Introduction

### Intergroup Contact and Empathy

There is a massive amount of research on the intergroup contact hypothesis (for meta-analyses see Pettigrew and Tropp, 2006, 2008). Regarding the mechanisms explaining how intergroup contact reduces prejudice, Pettigrew and Tropp (2008) conducted a meta- analysis of 54 studies on the three most studied mediators: enhancing knowledge about the outgroup (11 studies, 17 independent samples and 2,543 participants), reducing anxiety about intergroup contact (45 studies, 60 independent samples and 13,343 participants), and increasing empathy and perspective taking (9 studies, 14 independent samples and 2,362 participants). Their results revealed mediational effects for all three of these mediators, with emotional mediators (i.e., anxiety reduction and empathy) outperforming the mediation effect of increased knowledge.

Indeed, since then, empathy has increasingly received empirical attention in studies of intergroup relations showing that positive intergroup contact may enable one to take the perspective of outgroup members and empathize with their concerns, which may, in turn, contribute to improved intergroup attitudes.

Empathy has been conceptualized in different ways as a cognitive mechanism (role-taking or perspective-taking) enabling people to imagine the internal state of someone else (e.g., Borke, 1971) or as an emotional construct (affective empathy) enabling people to emotionally react toward other people’s experiences (e.g., Batson et al., 1987). In addition, empathy can be approached as dispositional or trait empathy (i.e., a personality-related characteristic; e.g., Davis, 1980; Konrath et al., 2011) or as situational or state empathy (i.e., negative and positive affects; e.g., Hein et al., 2018). Research on intergroup contact and empathy has largely been inspired by the affective state conceptualization suggested by Batson and his colleagues (Batson et al., 1987, 1997a,b, 2005). Specifically, in this paradigm, empathy consists of two distinct vicarious empathetic reactions, namely empathic interest and personal distress in a specific situation (Batson et al., 1987). While the latter refers to the egoistic motivation to reduce one’s own aversive arousal, the ultimate goal of the former is the reduction of the other’s need. For example, studies in different contexts have shown that affective empathy explains the effects of intergroup contact (especially quality) on adolescents’ intended bystander behavior among White British adolescents (Abbott and Cameron, 2014), positive attitudes toward diversity in organizations (Brouwer and Boros, 2010), helping intentions and increased commitment to help Black African Americans in a White American sample (Johnston and Glasford, 2018), and on the positive outgroup attitudes, perceived outgroup variability and less negative action tendencies among colored high school children in South Africa (Swart et al., 2011), to list just a few.

Moreover, the role of situational empathy is critical for intergroup contact intervention research, as inducing empathy for a member of a stigmatized group can improve attitudes toward the target and outgroup as a whole (Batson et al., 2002). Furthermore, a range of other emotional reactions associated with empathy, such as feelings of compassion (Batson et al., 1997b) or outrage at injustice (Dovidio et al., 2004) has been studied, supporting the critical role of the affective empathy in prejudice reduction (Dovidio et al., 2010).

Moreover, not only direct, but also extended contact (i.e., perceived intergroup contact) and CMC (computer mediated contact) or e-contact have been tested in terms of their potential to elicit empathy. The results are, however, less than straightforward. For example, in a study by Vezzali et al. (2017) among Italian and immigrant school children, extended contact had a positive effect on intergroup empathy, but only among those with a low or moderate level of direct contact. Similarly, in their study on e-contact between majority Australians and Indigenous Australians, Berry and White (2016) failed to find a direct association between e-contact and empathy. It is thus plausible that the effect of CMC or e-contact on online empathy depends on a number of contextual characteristics. Namely, there is research suggesting that e-contact may elicit online empathy and improve attitudes of dominant group members toward the non-dominant groups when text-based interactions are enriched with visual information (Grondin et al., 2019) or video interface (Bruneau and Saxe, 2012), or when there is an early disclosure of a stigmatized identity during text-based interaction (White et al., 2020). Moreover, it has been suggested that in CMC, empathy is closely linked to presence and so the higher immersion within a CMC channel may affect the perceived presence and empathetic ability experienced by participants (Nicovich et al., 2005). Similar conclusions about the contact effects online being moderated by the specific characteristics of online context and interaction have been reached in the meta-analysis of 23 studies on the effect of online contact on intergroup relations by Imperato et al. (2021). The authors suggested that future studies need to clarify for example how status equalization relates to empathy in computer-mediated communication. Thus, considering the limited evidence of text-based CMC or e-contact serving as an empathy machine in intergroup relations, in this study, we suggest looking at whether a VR contact characterized by a higher degree of immersion than any other forms of CMC contact may be used as a successful tool for increasing empathy.

### Virtual Reality and Empathy

One defining feature of VR is the high degree of immersion compared to two-dimensional animations such as conventional video games or text-based traditional CMC. Following Cummings and Bailenson (2016), immersion refers to “the extent to which the system presents a vivid virtual environment while shutting out physical reality” (p. 274). A system with a higher degree of immersion creates a stronger sense of presence in the user. Slater and Sanchez-Vives (2016) defines presence as “the illusion of “being there” in the environment depicted by the VR displays” (p. 5), while Lombard and Ditton (1997) characterize the construct as “the perceptual illusion of non- mediation.” Importantly, this strong sense of “being there” makes it possible to observe and measure people’s helping dispositions in the virtual world consistently with their self-reported empathy (Gillath et al., 2008) which can also be demonstrated with psychophysiological measures (D’Errico et al., 2020).

When designing interventions in VR, it is important to acknowledge that virtual environments are not simply backdrops for social interaction, but rather scenarios that allow for a wide range of interactions with features of the environment, increasing the so-called Plausibility Illusion (Psi): the illusion that the events happening in a virtual environment are actually *taking place* around the subject (Slater, 2009). This, together with the illusion of being there, increases the chances of realistic participant behavior, that is, behavioral reactions that they would have if the same social interaction happened in real life (Slater, 2009).

Overall, empathy-inducing interventions in VR can be classified into two categories: (1) via embodiment of an outgroup member or (2) interaction with an outgroup member. A recent review and meta-analysis of seven studies came to the conclusion that VR interventions that have participants take on outgroup members’ perspective can lead to increased empathy and perspective-taking (Ventura et al., 2020). Despite this encouraging message, a closer look at previous empirical studies using the embodiment of an outgroup member suggests somewhat mixed results. For example, a VR experience of discrimination from a racial minority perspective led to participants expressing increased empathy and understanding for the minority perspective (Roswell et al., 2020). In a somewhat different setting, experiencing schizophrenia symptoms with augmented reality similarly resulted in more empathy with people suffering from schizophrenia (Silva et al., 2017), especially in combination with an empathy inducing instruction (Kalyanaraman et al., 2010). A pilot study on enhancing empathy for individuals of a different gender (Muller et al., 2017) showed that most participants reported enhanced empathetic concern and perspective-taking after having experienced different scenarios of gender discrimination from a first-person perspective. In contrast, however, participants in Oh et al. (2016) study did not display increased empathy toward older people after having been embodied in an old avatar (Oh et al., 2016). Likewise, two studies by Tong and colleagues indicated that empathy toward people with chronic pain did not increase after embodying a chronic pain patient (Tong et al., 2017) or only on the *kindness* sub-scale of the questionnaire (Tong et al., 2020). The authors partially attributed this lack of an effect to the short duration of the intervention.

Scholars have also highlighted that embodying an outgroup member has the potential to change not only empathy, but also implicit attitudes toward said outgroup. Specifically, Peck et al. (2013) and Banakou et al. (2016) showed that embodying a Black avatar leads to greater decrease in implicit bias compared to embodying a White virtual body. Nevertheless, evidence in this regard is similarly mixed, as Groom et al. (2009) showed that implicit bias outside IVR increased after White participants embodied Black avatars. A recent account by Banakou et al. (2020) suggests that affects may have a central role in this process, since implicit attitudes toward Black people worsened in subjects that experienced negative affection during embodiment of a Black avatar. Similarly, Hasler et al. (2017) found that the effect of embodiment of an outgroup avatar on implicit bias is moderated by the likability of outgroup interaction avatar. Lastly, Patané et al. (2020) showed that the positive effect of embodiment of an outgroup avatar on implicit bias could be strengthened by carrying out collaborative tasks. Finally, when discussing such perspective taking interventions, it is important to note that taking someone else’s perspective can both diminish and further reinforce intergroup stereotypes and negative bias instead of mitigating it when presented with information confirming one’s stereotype toward a social group (Skorinko and Sinclair, 2013).

A smaller set of studies used the intergroup contact paradigm in VR. Three studies compared a 360 degree video with a 2D version of the same material regarding its potential to elicit empathy toward the outgroup target. Seeing a 360 degree video of a man telling about his life with schizophrenia, did not lead participants to report more empathy with schizophrenic patients than in a normal video or in a control condition (Stelzmann et al., 2021). In Schutte and Stilinović’s (2017) study, a refugee girl guided participants through the video, and the results showed that the more immersive 360 degree version led to higher empathy than the 2D version and this effect was mediated by the degree of engagement. Similarly, in a study by Christofi et al. (2020) participants saw animated scenes from a drug user’s life either in VR or on a desktop screen. Their results showed no difference in the average related levels of empathetic concern for drug users between conditions. However, within the VR condition, participants reporting a higher sense of body ownership and agency over the avatar representing the drug user in the VR animation reported higher levels of empathy Christofi et al. (2020). Further, Hasler et al. (2014) showed that Jewish-Israeli participants who conversed with an outgroup avatar (Palestinian) reported increased empathy and understanding of the outgroup position, especially if the avatar mimicked the participant.

Summarizing these findings and taking into account the commonly small samples, the evidence thus far seems mixed. To what degree and under what circumstances VR interventions can increase empathy toward different outgroups defined by different health status, ethnicity, gender, or other social categories remains a relatively open question. The vast majority of studies examining effects of VR on empathy used paradigms that embodied participants in an avatar representing an outgroup member, directly presenting their perspective. As much as this can result in increased empathy toward the outgroup target, it can also produce distress and have detrimental effects on outgroup attitudes (Banakou et al., 2020). Much less is known about first-hand contact with virtual outgroup members when embodying a self-representing avatar. Previous studies representing the contact paradigm seem to suggest that deep immersion into the virtual world can lead to a stronger sense of presence and this supports the desired effects. Barbot and Kaufman (2020) also question the importance of the content of the VR interventions: in their study, participants’ empathy increased after using different VR applications regardless of the specific media content. The main factor contributing to the increase in different facets of self-reported empathy was, in their study, users’ experience with VR, and especially illusion of body ownership and agency.

In this study, we argue that due to its ability to produce the experience of co-presence, direct VR intergroup contact may have the potential to affect empathy without empathy inducing instructions. Namely, as seen from the reviewed research above, previous experimental studies on the link between empathy and helping behavior typically manipulated or stimulated empathy toward a person in need by perspective-taking instructions. The experimental investigations of empathy in intergroup contexts, including CMC and VR contact, have typically used somewhat similar designs, in which the participants were exposed to instances of discrimination or other negative events experienced by an out-group member and were then asked to imagine how they felt during the task, after which their attitudes toward the out-group were assessed. This approach has a range of serious limitations that the design of the present study circumvents. Firstly, not only the altruistic nature of the behavior produced in such designs has been contested, but also as Neuberg et al. (1997) argued in their critique, empathic concern should similarly arise from the mere perception or engagement in social interaction with someone in need or disadvantage. Secondly, as Vorauer and Sasaki (2009) have criticized, this approach has little ecological validity as individuals are not placed in an actual interaction situation. Their study showed that due to the activation of metastereotypes, adopting an empathic stance toward out-group members was beneficial outside of, but not within, intergroup-contact situations, in which individuals’ perspective-taking efforts were egocentric and counterproductive. Thirdly, as previous research has mostly used a retrospective assessment of empathetic concerns during message processing (Shen, 2010), the extent to which empathy experienced during the specific situation characterizes the emotional states after the situation remains unclear. Lastly, as stressed by Verkuyten (2004), intergroup and power relations are situation- and context-specific and so dominance or disadvantage should not be automatically related to particular social categories as their nature and extent change from one situation to another. Therefore, studies on the intergroup outcomes of contact should not automatically attribute social disadvantage to minority group members, but let the contact experience modify intergroup stereotypes and boundaries.

## The Aim and Research Questions of the Study

In this study, we explore the potential of direct positive intergroup VR contact to affect situational affective empathy (i.e., to reduce personal distress and increase empathic interest), when no perspective-taking instruction is introduced. This way, we look at the VR contact as a means to induce individuals’ general ability to empathize with another (McLaren et al., 2019) following positive intergroup contact. This is also reflected in the measure of empathy used in this study that assesses personal distress and empathic feelings at the moment of replying (rather than a retrospective report of empathy experienced during the interaction) and without a specific target. Specifically, we let participants experience positive (collaborative) direct intergroup VR contact (AltspaceVR),^1^ which satisfies the criteria of optimal contact as defined by Allport (1954) and test the emotional consequences of such contact.

Considering the lack of research on intergroup relations using commercial, freely available VR platforms such as AltspaceVR and the controversial results of previous studies reviewed above, we set two exploratory research questions:

RQ1: To what extent does the Altspace virtual environment enable perceived body ownership and control over the avatar and co-presence with other avatars that are present in the virtual environment?

RQ2: Are there differences in the effects of positive (collaborative) VR intergroup contact (i.e., interaction with an avatar representing the racial outgroup) vs. ingroup contact (i.e., interaction with an avatar representing the racial ingroup) on different facets of empathy?

## Materials and Methods

### Procedure

Participants filled in a pre-test questionnaire online using Psytoolkit (Stoet, 2010, 2017), then arranged a time to take the VR experiment at Aalto University (Espoo, Finland). The pre-test included demographic data and several baseline measures. The whole procedure took around 20 min. The data collection took place between May and November 2021. The questionnaire also included an informed consent form to check in order to take part in the experiment. They then took the VR experience 3–7 days later. In case of underage participants, a lack of objections from their legal guardians was required in order to take part to the experiment. The participants themselves additionally had to check the informed consent form.

**FIGURE 1:**
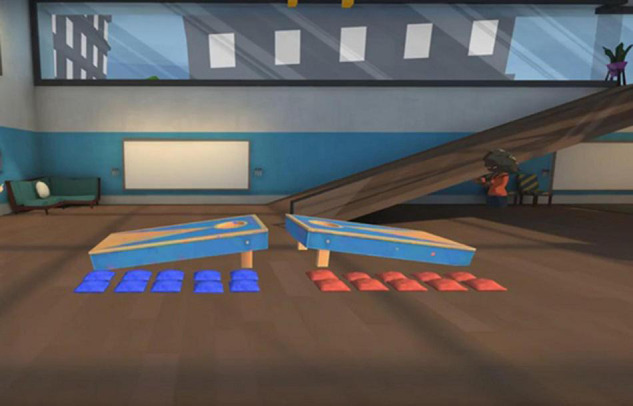
The virtual environment where participants play the throwing game. Screenshot with permission from https://altvr.com.

**FIGURE 2:**
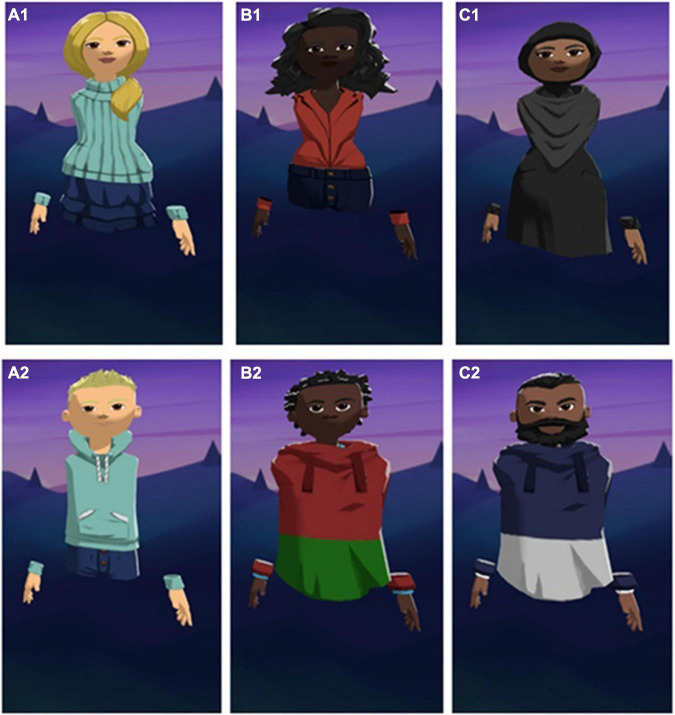
Avatars representing the ingroup (control condition, **A1,A2**), the primary outgroup (experimental condition **B1,B2**), and the secondary outgroup (all conditions, **C1,C2**). Screenshot with permission from https://altvr.com.

All subjects underwent the experiment in laboratory settings. Participants were instructed by the experimenter on the use of the Oculus headset and helped in adjusting it, if needed. If they wore glasses, an adapter was added to the device. Then, participants started the Altspace tutorial to learn how to interact with the environment and steer their avatar. Once the tutorial was completed, they were instructed to create an avatar that resembled them. All participants followed this instruction. After freely editing their own avatar, participants embodied it for the whole duration of the experience. This was done for two reasons: first, it should result in a stronger sense of body ownership of one’s own avatar and self-relevance of the experience; and second, it should create the expectation that the other player in the game did the same and therefore is a member of the group represented by the other avatar (see below). Subsequently, the experimenter led them to the first virtual environment, that can be seen in Figure 1. The experimenter was also present in this environment, steering an avatar (see Figure 2) from a desktop computer in a different location. The confederate’s avatar was in mute mode, meaning that verbal exchange was not possible. It was possible to interact through gestures (e.g., waving at each other).

Upon entrance, the participant was presented with the following instruction text: “We now ask you to play ball toss with another participant in the experiment, that is playing simultaneously from another location. You and your partner belong to the SAME TEAM and need to score 10 points by throwing the red and blue bags through the hole on the board (1 point = bag through the hole). You can find the bean bags next to the game board. You and your partner are supposed to TAKE TURNS at throwing. The game is over once you have scored a total of 10 points as a team. Another team is currently playing or will shortly begin to play in another room. The team that will score 10 points first will be the winner. Please, we ask you to respect TURN TAKING with your partner. The game starts when you are ready. Please, position yourself on the opposite side of the other player. YOU throw first. The experimenter informs when you have reached 10 points. Good luck!” Shortly after, a third avatar steered by a confederate with an Oculus headset would join the room.

**FIGURE 3:**
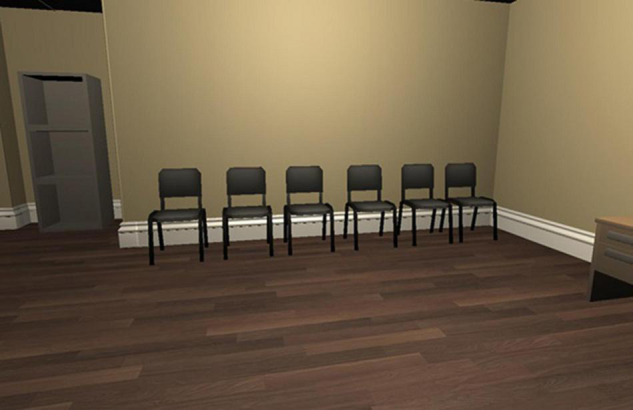
The virtual environment waiting room. Screenshot with permission from https://altvr.com.

Depending on the condition the participants were randomly assigned to, they would play a throwing game with either a white (control) or a black (experimental) avatar. Participants were randomized using Research Randomizer 4.0 (Urbaniak and Plous, 2013). Once the participant and the confederate scored 10 points as a team, they were stopped by the experimenter. The participant was then instructed to join a separate virtual room (see Figure 3) while the team scores were computed. There, they would read the following instruction text: “The other team is still playing or we are assessing scores and game time. Please, take a seat while we set the next stage of the experiment. We will let you know the results as soon as possible.” Another avatar steered by the confederate on a desktop computer would be sitting on the farthest seat. Regardless of the condition, this avatar was designed to resemble Middle Eastern physical features (see Figure 2). Participants would spend 3–5 min in that room, to ensure they had the time to gain confidence with the new environment and take a seat. As in the previous phase, interaction between avatars was only possible through gestures. Then, the participant was told that their team had won, and that they could remove the headset once ready.

Subsequently, the participant took a post-test questionnaire on a desktop computer, which included post-exposure measures and different manipulation checks (see “Measures” section for details). Furthermore, they were asked to give their feedback on the VR experience. Following the post-test questionnaire, participants were debriefed about the purpose of the study.

Each participant received a 10EUR gift card upon completion of the study as a compensation, either via email or in the lab.

### Virtual Reality Implementation

Participants completed the experiment at [blinded location], using an Oculus Quest 2 headset. This head-mounted display has a resolution of 1,832 × 1,920 pixels per eye, which can be displayed at up to 120 Hz. It weighs 503 grams and can adjust interpupillary distance by sliding the lenses in three different positions. The headset and controllers are equipped with inside-out tracking and allow 6 degrees of freedom head and hand tracking. Those features enable orientation and positional tracking in the virtual space, as well as integration of virtual hands (see https://developer.oculus.com/resources/oculus-device-specs/ for more technical specifications). The remaining two participants (*n* = 2) completed the experiment using their own VR headset, given that AltspaceVR was supported. The accepted devices were Oculus Rift, Rift S, Quest, Quest2, and HTC VIVE. More information about the part of the sample using their own VR devices can be found in the participants section. Since they belonged to the pilot experiment, their data is not included in the analyzed sample.

The virtual environment was partly created using the Altspace built-in tools and partly modeled on the software Unity3D (version 2020.3.9f1), to be later uploaded to AltspaceVR. AltspaceVR is a free social app that is available on most VR headsets. Its main purpose is to allow people to gather, interact, and cooperate in VR. It allows users to organize and hold events such as shows, meetings, workshops, and so on, as well as to create highly customizable virtual environments where it is possible to organize large gatherings. Avatars are also highly customizable and allow both voice interaction and text messaging. Altspace was chosen to support this experiment for two main reasons. Firstly, its completely customizable features allow for potentially creating any kind of environment, be it a crowded event, a virtual lab, or a remote extraterrestrial location. Secondly, it is accessible to most VR users worldwide, and being already widespread it allows for studying social processes that might already be taking place between users in this environment, and to implement interventions that can be delivered to an already existing population.

**FIGURE 4:**
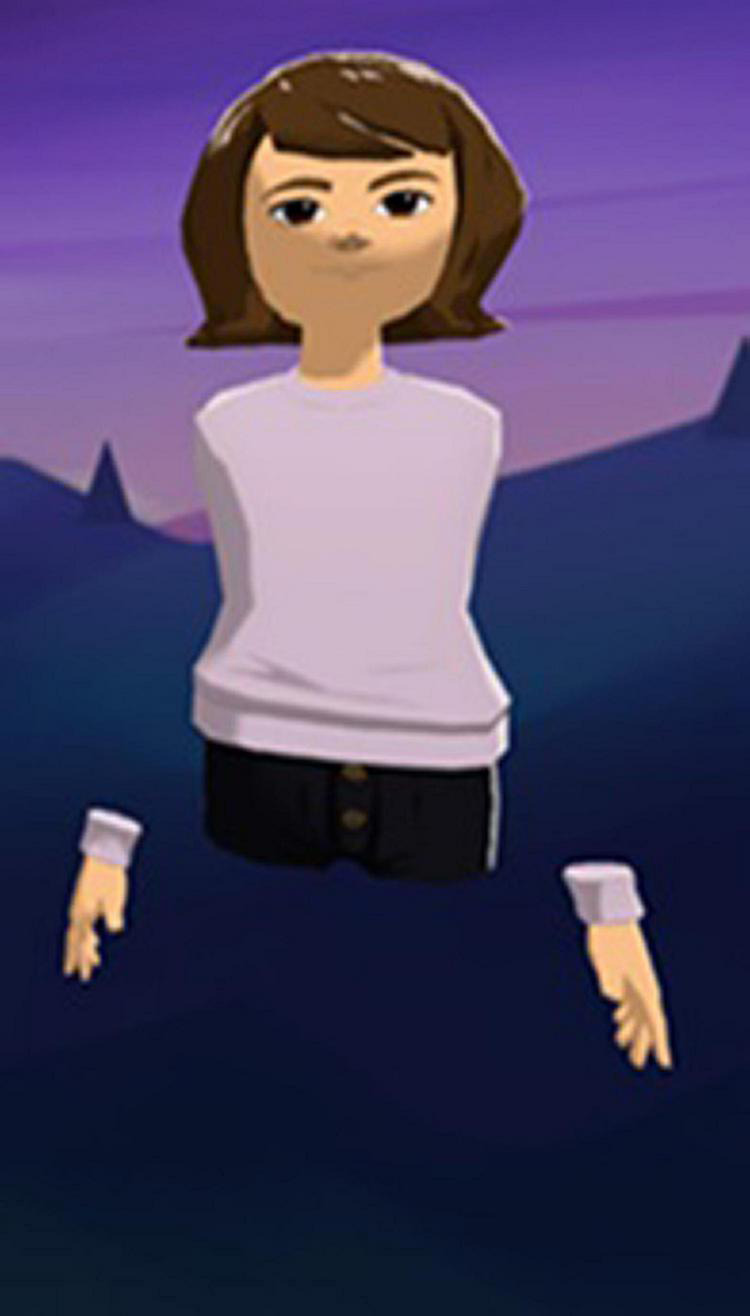
Avatar steered by the experimenter across all conditions. Screenshot with permission from https://altvr.com.

The avatars used by confederates were entirely modeled in Altspace. Participants assigned to the experimental condition interacted with an avatar representing a person of African ethnic background, while participants assigned to the control condition played with avatars representing a person of Caucasian ethnicity. Regardless of the assigned condition, all participants also briefly faced an avatar representing a person of Middle Eastern ethnic background (exception made for *n* = 3, due to difficulties in loading the virtual environment). Each participant only interacted with avatars of the same gender they identified with, to avoid effects specific to female-male interactions. In total, 6 avatars were designed to interact with participants. Those can be seen in Figure 2. A confederate was also constantly present in the virtual environment, to provide support and guide participants through the experiment. The confederate’s avatar can be seen in Figure 4 and did not vary across participants.

### Participants

A total of *N* = 64 participants took part in the experiment. Among those randomly assigned to the experimental condition (n1 = 32), the average age was 20 years, and they had none to little previous experience with VR (m1 = 2.03/5). Participants in the control condition (n2 = 32) were on average 17 years old. The age difference between groups stems from changes in the recruitment strategy as we started targeting high school students after our initial university student sample. Similarly to the experimental group, they had none to little previous VR experience (m2 = 2.09/5).

Twenty nine of the 33 participants in the control condition indicated being of white ethnicity with four participants additionally indicating being of Asian ethnicity. One participant in this condition indicated having an African background. We excluded this participant’s data from all analyses, leaving us with 32 participants in the control condition. In the experimental condition, all 32 participants indicated to be of White ethnicity with one participant additionally self-identifying as of Asian ethnicity.

### Measures

All scales were translated to Finnish by two independent researchers.

#### Empathy

Batson et al. (1987) affective empathy scale was used to measure self-reported levels of situational empathy. This instrument is composed of fourteen adjectives describing different emotional states and the participants are asked to what extent they experience these emotions on a scale from 1 = “not at all” to 7 = “very much.” The items form two subscales, personal distress (alarmed, embittered, annoyed, uncomfortable, baffled, embarrassed, worried, upset) and empathic interest (empathetic, sensitive, affable, compassionate, affectionate, moved). The measure can be used as either a difference score that is computed by subtracting the distress scale score from the overall score as originally suggested by Batson et al. (1987), or as a two-dimensional scale with separate scores for personal distress and empathic interest following an interaction situation. In this study, we decided to report separate scores for both sub-scales to test the possible effect of VR contact on both components of empathy. Crohnbach’s alpha for the different empathy scales were satisfactory (0.88 and 0.86 for empathic interest pre-and post-intervention, respectively; 0.76 and 0.82 for personal distress pre-and post- intervention, respectively). It is worth noting that this scale provides a reliable measure of situational empathy rather than dispositional, thus it is highly influenced by the context and not stable over time. We measured empathy first during the pre-test, namely 3–7 days prior to the VR experience, with the intent of having a baseline level of empathy, while the post-test measurement took place immediately after taking the experiment.

#### Body Ownership and Perceived Control Over the Avatar

A measure of body ownership from Peck et al. (2013) was used to assess the degree to which participants identified with the avatar they steered in the virtual environment on a five-point Likert scale, where 1 = “strongly disagree” and 5 = “strongly agree.”

Specifically, the items “I felt as if the body I saw in the virtual world might be my body” (item 1) and “I felt like the avatar was not me” (item 4) refer to the degree of self-overlap with the virtual body and are combined to form an indicator of body ownership by calculating (Item1 + 6 - Item 4)/2. The correlation between the two items was –0.59. The item “I felt like I controlled the avatar as if it was my own body” was used as a single-item measure of control over the avatar.

#### Co-presence

A four-items scale measuring co-presence in VR was used to measure the degree of salience of the other person in the interaction (Short et al., 1976), with particular reference to Biocca and Harms’s (2002) notion of *co-presence of the embodied other* as “the detection and awareness of the co-presence of others’ mediated body.” Items (adapted from Biocca and Harms, 2002) were rated on a five-points Likert scale from 1 = “strongly disagree” to 5 = “strongly agree.” Example items are “I often felt as if my partner and I were together in the same room,” “I hardly noticed my partner in the room.” The Crohnbach’s alpha for this scale was satisfactory, α = 0.76.

### Online Pilot Study

To check whether the application produced a sufficient feeling of body ownership of the avatar and of co-presence with the other avatar, we tested the procedure with three participants online who used their own VR headsets. Procedures were slightly different for these participants. First of all, participants’ eligibility was checked depending on the device they owned. Upon sign-up for the VR experiment, they were sent an email with preliminary instructions to download the Altspace app on their device before the chosen date. Then, 30 min before the experiment was set to begin, they were sent the full instructions via email, including username and password to access a pre-made account with access to the virtual environment. The experimenter was present in the throwing game room to guide through the experience. Our three online pilot participants reported an average score 4 on body ownership and 3.67 on co-presence, indicating that the procedure worked sufficiently well.

Nevertheless, due to the differences in procedure that may have hindered the generalizability of results, it was decided not to include the pilot data in the final sample.

### Data Analysis

We used independent *t*-tests for testing differences between conditions in co-presence, body ownership, and perceived control over the avatar. The two subscales of the empathy scale (empathic interest and personal distress) as well as the difference score between general empathy and distress were analyzed with a mixed ANOVA using condition as a between- and time as (before the VR session vs. after) as the within-subjects variable. We further visually inspected relations between variables of interest for exploratory analyses.

All analyses were conducted in R software (R Core Team, 2017) with the following packages: *rstatix* (Kassambara, 2021), *tidyverse* (Wickham et al., 2019), *dplyr* (Wickham et al., 2020), *forcats* (Wickham, 2021), *ltm* (Rizopoulos, 2006), *corrgram* (Wright, 2021), *stargazer* (Hlavac, 2018), *ggplot2* (Wickham, 2016, p. 2), *cowplot* (Wilke, 2020), apaTables (Stanley, 2018), and *Routliers* (Delacre and Klein, 2019).

## Results

Of the 32 participants in the control condition, 31 indicated that they thought the avatar they interacted with was Finnish. Two participants perceived the avatar as being homosexual, one participant thought of the avatar being mentally disable, and one thought him to be an immigrant. In the experimental condition, 24 out of 32 participants chose “African background,” 4 “Arabic,” 13 “Finnish,” 1 “homosexual,” 3 “migrant,” and 5 “Muslim.” Such information was collected through a single-item measure asking participants to check which social groups they thought the avatar belonged to. This was encompassed in the post-test questionnaire as a manipulation check. The majority of participants thus interpreted the manipulation of the avatar as intended.

**FIGURE 5:**
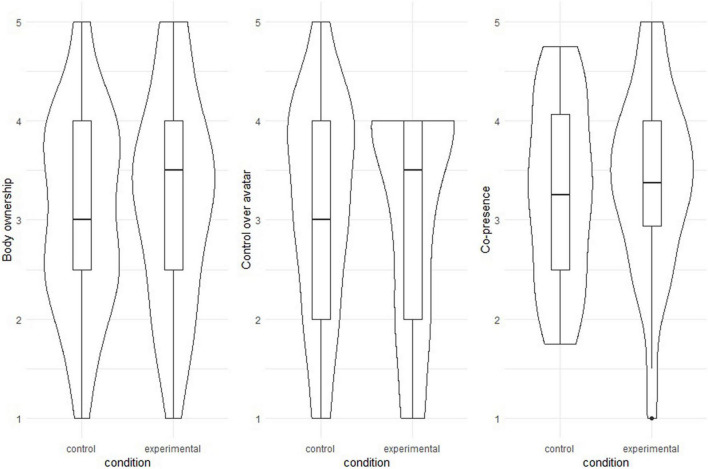
Violin and boxplots for the following variables: body ownership, perceived control over the avatar, and feelings of co-presence (from left to right). Plotted variables are grouped by condition. Lines within the boxes indicate the median, with the upper and lower limit representing the 75th and 25th percentile, respectively. Body ownership was calculated as a mean score of two items, perceived control over the avatar (measured with a single item), and feelings of co-presence (calculated as a mean score of four items). Measurement scales ranged from 1 to 5.

Overall values for body ownership (*m* = 3.14, *sd* = 1.02), co-presence (*m* = 3.33, *sd* = 0.91), and perceived control over avatar (*m* = 3.09, *sd* = 1.09) were above the middle of the scale. Conditions did not differ significantly regarding body ownership (*m*(control) = 3.08, *sd*(control) = 0.99, *m*(experimental) = 3.20, *sd*(experimental) = 1.05; *t* = –0.49, df = 61.78, *p* = 0.63), perceived control over the avatar (*m*(control) = 3.13, *sd*(control) = 1.10, *m*(experimental) = 3.06, *sd*(experimental) = 1.11; *t* = 0.23, df = 62.00, *p* = 0.82) or co- presence (*m*(control) = 3.27, *sd*(control) = 0.91, *m*(experimental) = 3.38, *sd*(experimental) = 0.93; *t* = –0.48, df = 61.97, *p* = 0.63). The distributions of body ownership, perceived control, and co-presence variables for both conditions are shown in Figure 5.

**FIGURE 6:**
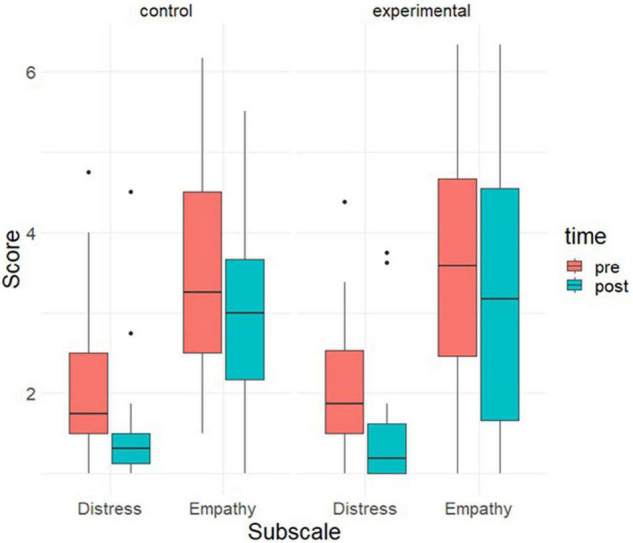
Boxplot for the sub-scales of the empathy scale divided by measurement timepoint and condition. The lines within the boxes indicate the median with the upper and lower limit indicating the 75th and 25th percentile, respectively.

The results of the mixed between-within ANOVA indicated a main effect of time on both empathic interest [*F*(1, 62) = 9.72; *p* = 0.003; η^2^ = 0.023] and personal distress [*F*(1, 62) = 41.46; *p* < 0.001; η^2^ = 0.156], but not for the difference score calculated as the difference between empathic interest and personal distress [*F*(1, 62) = 2.66; *p* = 0.11; η^2^ = 0.006]. As can be seen in Figure 6, the scores for both empathic interest and personal distress decreased from before pre- to post-intervention, to a similar degree in both the experimental and control group. Removing participants who did not attribute an African background to the avatar in the experimental group did not change the pattern of results. We also detected three outliers in personal distress at baseline and four outliers at follow-up. Removing them from the mixed ANOVA for personal distress scores did not change the pattern of results.

We inspected correlations between the empathy variables and the other analyzed variables for exploratory purposes. Without any preset hypothesis, our purpose was to explore the relationship between empathy and measures related to embodiment and contact in VR (i.e., body ownership, co-presence, and control over the avatar). We found that the empathy difference score measured after the VR experience correlated significantly with feelings of body ownership (*r* = 0.34, *t* = 2.89, *df* = 62, *p* = 0.005) and control over the avatar (*r* = 0.35, *t* = 2.90, *df* = 62, *p* = 0.005) across groups. In other words, the more participants had an experience of control over and body ownership in their avatars, the higher they scored in general empathy (i.e., difference score) after the intervention. One noteworthy pattern that warranted further inspection was that empathic interest after the intervention highly correlated with scores on the co-presence scale, but only in the experimental condition (*r* = 0.48, *t* = 3.00, *df* = 30, *p* = 0.005). We followed up on this by conducting a regression predicting post-intervention empathy scores by condition (experimental vs. control), co-presence scores, and their interaction. The interaction effect was significant (*b* = 0.87, *t* = 2.48, *p* = 0.016), indicating that the relation between co-presence and empathic interest differed between conditions. No further differences between the two conditions studied were detected. Supplementary Table 1 shows means and standard deviations for all variables of interest as well as the correlations between them across the whole sample.

## Discussion

In the current study, participants immersed themselves into a virtual world of intergroup relations through a commercial, freely available VR application, “AltspaceVR,” using head-mounted displays. We did not find that our positive, collaborative intergroup contact intervention would have a significant effect on participants’ empathy when compared to the effect of an ingroup contact in the control group. While both experienced distress and empathic interest were lower after the VR session compared to pre-test scores, this was true to a similar degree for both groups of participants.

The failure to obtain the effect of intergroup contact may be due to several reasons. Firstly, it is important to point out that our measure of empathy was, unlike in many earlier studies, not specifically related to intergroup situations nor was it a *post hoc* measure of empathy experienced during the interaction situation. Rather, it was a measure of how empathetic and distressed participants felt at the moment of answering and thus represented a general situational affective empathy. At the first measurement timepoint, this was outside of any specific context, with the intention of measuring baseline levels of empathy outside any social interaction; at the second measurement timepoint, this was right after the VR experience and is thus most likely mainly influenced by that experience. Any interpretation of our results rests on this conceptualization and measurement of empathy. The fact that participants in both groups reported relatively low levels of distress after using AltspaceVR shows that the VR experience was not perceived as negative and did not elicit feelings of anxiety or discomfort in most participants.

Secondly, we are inclined to suggest that increased empathy following intergroup contact should not automatically be perceived as a desired effect. Indeed, previous research shows that intergroup contact is most likely to lead to increased empathy, but it does so when the target outgroup member is perceived as disadvantaged or in need. A review by Christofi and Michael-Grigoriou (2017) shows that most studies aimed at increasing empathy in VR emphasize the disadvantaged status of the contacted outgroup, thus building on the unequal status of the interacting agents. This was not the case in the current study: the outgroup avatar was at the same skill level as the ingroup avatar in the control group and at a similar skill level as the participants’ own avatars regarding the task at hand (the tossing game) and therefore probably did not elicit the impression of being disadvantaged or in need. We consider this a feature of our design rather than a bug as equal status of intergroup partners is one of the main conditions for intergroup contact to decrease prejudice as postulated by Allport (1954). Actually, Imperato et al. (2021) found that in all 23 studies included in their meta-analysis, online interaction was characterized by equal status of interaction partners. It thus seems to be a rather universal feature of online interaction emphasizing the potential of online contexts as a platform for prejudice reduction. Relatedly, participant instructions said they were engaging in a competition with another team. This competition situation might have led to a decrease in empathic interest (Zaki, 2014), especially since the empathy measure did not indicate a specific target for these feelings. This also adds to the discussion on the role of empathy in intergroup contexts in general as intergroup contact has been shown to have ironic effects leading to more helping behavior and paternalistic attitudes, which happens at the expense of willingness to combat prejudice and inequality (Nadler, 2016).

Thirdly, a competing line of interpretation relies on the age of the sample, since most of the participants were high-school students. Indeed, a meta-analysis by Konrath et al. (2011) suggests that dispositional empathy is steadily decreasing, especially in younger generations. One of the possible explanatory paths lies in the growth of online social interaction and use of social media platforms at the expense of real-life interactions, suggesting that younger generations that make wider use of those may have reduced capacities of perspective- taking and empathic concern above all. This constitutes a hypothetical limitation of the current study, which would ideally be replicated with an older sample.

Lastly, the chance that avatar features hindered the creation of solid group memberships among the two conditions also needs to be taken into account. Indeed, a potential failure to represent and differentiate outgroup and ingroup members could have undermined the effectiveness of the virtual intergroup contact experience.

We are aware that any theoretical interpretation based on the lack of statistical effects can only be a tentative explanation. Thus, we wish to specify that all lines of thought are purely speculative premises to the failed increase in empathy following the experimental manipulation. When it comes to potential moderators of VR contact, we focused on factors related to the degree of immersion of the experience. We could show that a relatively simple, freely available solution like AltspaceVR led to acceptable average levels of body ownership, control over the avatar, and feelings of being in the same room as the interaction partner (co-presence). It must be noted, however, that this interpretation is merely based on the means of the scales used to measure participants’ experiences with no validated cut-off points for “sufficient” levels of different aspects of immersion. Second, the current sample had a low level of previous experience with VR and this “novelty effect” might wear off with repeated use. More experienced users of VR might not find the rather simple design of AltspaceVR convincing. The fact that most participants identified the group membership of our avatars as intended shows that such manipulations of avatar ethnicity are possible in this environment.

We also explored the relationship between our post-intervention empathy measures with the above-mentioned potential moderators. Our exploratory analyses showed no clear patterns for the relationship between body ownership or control over the avatar and empathic interest or personal distress among participants after the intervention. This is in contrast with recent findings by Barbot and Kaufman (2020), who showed that the illusion of body ownership and agency were the strongest predictors for different measures of empathy, regardless of the content of the VR experience. However, we did find significant correlations between the empathy difference score as suggested by Batson et al. (1987) and both body ownership and perceived control over the avatar in both groups’ post-test scores. In other words, the more participants had an experience of body ownership and control over their avatars, the higher they scored in general empathy after the intervention. This pattern of results and the lack of differences between the groups regarding empathy, is in line with the results from that study.

Moreover, we did observe a tentative but potentially interesting pattern for co-presence: participants who felt a stronger sense of being in the same virtual environment as the other avatar tended to report more empathic interest after the VR experience.

Interestingly, this correlation was only significant in the experimental group, meaning that empathy correlated with feelings of co-presence with an outgroup avatar, but not with one belonging to the ingroup. It is worth pointing out that there was no significant difference in terms of co-presence across conditions, which leads to exclude the chance of this correlation being due to a hypothetical increased awareness of the other avatar when this belongs to an outgroup. This result was reaffirmed by the interaction finding suggesting that the effect of intergroup contact on empathy in VR is moderated by feelings of co-presence with an outgroup avatar. Not only is this explanation in line with the notion that a strong sense of presence supports the desired effects of VR interventions (Barbot and Kaufman, 2020), but it further suggests that the effect of VR intergroup contact on empathy is produced by the experience of co-presence with an outgroup avatar. In other words, VR contact can be employed to make a user “truly” experience positive contact with outgroups in the VR environment resulting in greater empathy. As these analyses were exploratory and *post hoc* we want to emphasize that this interpretation is very tentative and hypothesis-generating at best.

## Conclusion

Early evidence shows that there is great prevalence among empathy-enhancing VR experiments of designs that enact embodiment in an outgroup member to create empathy through perspective-taking (Christofi and Michael-Grigoriou, 2017). In the current design, we aimed at recreating intergroup contact without requiring participants to imagine how they would feel in someone else’s shoes. As Lara and Rueda (2021) point out, “a distinctive feature of the in-her-shoes virtual embodiment is its high likelihood of giving rise to an “empathic concern,” that is, greater compassion and interest for those suffering from negative experiences and a greater willingness to alleviate it” (p. 3). According to the authors, it is of primary importance for this purpose to underline that VR offers the chance of “imagining-other” rather than attempting at being-other, because here lies the meaning of empathic concern. This study explored the potential of VR to increase empathy through equal, ingroup-embodied intergroup contact, about which little is known so far. Indeed, the importance of VR in terms of harvesting empathy through perspective-taking and empathic concern, when it comes to the mechanisms of embodiment and personal distress, has been so far emphasized with specific regards to the prevalence of designs building on the status of disadvantage of minorities.

The current study showed that both empathic interest and personal distress were lower after the VR experience in both the ingroup and outgroup contact condition, and tentatively suggests that situational empathy may be influenced by intergroup interaction in VR with an equally agentic outgroup member via the experience of co-presence. However, it is of primary importance to stress the speculative nature of such hypothesis, given the lack of statistically significant effects. Further research would, however, be needed to investigate changes in terms of intergroup empathy and other intergroup outcomes as well as the role of other features specific to VR in explaining the effects of VR contact.

## Data Availability Statement

The raw data supporting the conclusions of this article will be made available by the authors, without undue reservation.

## Ethics Statement

The studies involving human participants were reviewed and approved by Aalto University. Written informed consent from the participants’ legal guardian/next of kin was not required to participate in this study in accordance with the national legislation and the institutional requirements.

## Author Contributions

MT, IJ-L, and MBA contributed to conception and design of the study. MT collected the data. MBA performed the statistical analysis. MBA, IJ-L, and MT wrote the first draft of the manuscript. All authors contributed to manuscript revision, read, and approved the submitted version.

## Conflict of Interest

The authors declare that the research was conducted in the absence of any commercial or financial relationships that could be construed as a potential conflict of interest.

## Publisher’s Note

All claims expressed in this article are solely those of the authors and do not necessarily represent those of their affiliated organizations, or those of the publisher, the editors and the reviewers. Any product that may be evaluated in this article, or claim that may be made by its manufacturer, is not guaranteed or endorsed by the publisher.

## Footnotes

1
https://altvr.com/
