# Supplementary material for: Investigating the Influence of Intergroup Contact in Virtual Reality on Empathy: An Exploratory Study Using AltspaceVR
Source: Front Psychol. 2022 Feb 2;12:815497. doi: 10.3389/fpsyg.2021.815497 (PMC8848353; doi:10.3389/fpsyg.2021.815497)
Supplement: Supplementary file 1 [file Table_1.DOC]

Table 1 
 
Means, standard deviations, and correlations with confidence intervals
 
Variable	M	SD	1	2	3	4	5	6	7	8	
											
1. Co-presence	3.33	0.91	 	 	 	 	 	 	 	 	
 	 	 	 	 	 	 	 	 	 	 	
2. Body ownership	3.14	1.02	.30*	 	 	 	 	 	 	 	
 	 	 	[.06, .51]	 	 	 	 	 	 	 	
 	 	 	 	 	 	 	 	 	 	 	
3. Control over body	3.09	1.09	.24	.42**	 	 	 	 	 	 	
 	 	 	[-.00, .46]	[.20, .61]	 	 	 	 	 	 	
 	 	 	 	 	 	 	 	 	 	 	
4. Empathic interest T1	3.54	1.40	.07	.23	.19	 	 	 	 	 	
 	 	 	[-.18, .31]	[-.02, .45]	[-.05, .42]	 	 	 	 	 	
 	 	 	 	 	 	 	 	 	 	 	
5. Personal distress T1	2.07	0.84	-.13	-.06	-.27*	.14	 	 	 	 	
 	 	 	[-.37, .11]	[-.30, .19]	[-.49, -.03]	[-.11, .37]	 	 	 	 	
 	 	 	 	 	 	 	 	 	 	 	
6. Empathy difference score T1	1.47	1.52	.14	.24	.33**	.84**	-.42**	 	 	 	
 	 	 	[-.11, .37]	[-.01, .46]	[.09, .53]	[.75, .90]	[-.60, -.20]	 	 	 	
 	 	 	 	 	 	 	 	 	 	 	
7. Empathic interest T2	3.13	1.36	.27*	.31*	.23	.70**	-.04	.66**	 	 	
 	 	 	[.03, .48]	[.07, .51]	[-.01, .45]	[.55, .81]	[-.28, .21]	[.50, .78]	 	 	
 	 	 	 	 	 	 	 	 	 	 	
8. Personal distress T2	1.43	0.65	-.00	-.15	-.31*	-.07	.46**	-.31*	.01	 	
 	 	 	[-.25, .25]	[-.38, .10]	[-.52, -.07]	[-.31, .18]	[.24, .63]	[-.52, -.07]	[-.23, .26]	 	
 	 	 	 	 	 	 	 	 	 	 	
9. Empathy difference score T2	1.69	1.50	.24	.34**	.35**	.67**	-.23	.74**	.90**	-.42**	
 	 	 	[-.00, .46]	[.11, .54]	[.11, .55]	[.50, .78]	[-.45, .01]	[.60, .83]	[.84, .94]	[-.60, -.20]	
 	 	 	 	 	 	 	 	 	 	 	

Note. M and SD are used to represent mean and standard deviation, respectively. Values in square brackets indicate the 95% confidence interval for each correlation. The confidence interval is a plausible range of population correlations that could have caused the sample correlation (Cumming, 2014). * indicates p < .05. ** indicates p < .01.
